# Quality assurance and reporting for FLASH clinical trials: The experience of the FEATHER trial

**DOI:** 10.1002/mp.18100

**Published:** 2025-09-03

**Authors:** Isabella Colizzi, Robert Schäfer, Jonas Brückner, Gaia Dellepiane, Martin Grossmann, Maximilian Körner, Antony John Lomax, David Meer, Benno Rohrer, Carla Rohrer Bley, Michele Togno, Serena Psoroulas

**Affiliations:** ^1^ Center for Proton Therapy Paul Scherrer Institut Villigen PSI Switzerland; ^2^ Department of Physics ETH Zurich Zurich Switzerland; ^3^ Clinic for Radio‐Oncology and Medical Oncology Small Animal HospitalVetsuisse Faculty , University of Zurich Zurich Switzerland; ^4^ Department of Radiation Oncology University Hospital Zurich (USZ) University of Zurich (UZH) Zurich Switzerland

**Keywords:** clinical trial, FLASH, proton therapy, ultra‐high dose rates

## Abstract

**Background:**

Research on ultra‐high dose rate (UHDR) radiation therapy has indicated its potential to spare normal tissue while maintaining equivalent tumor control compared to conventional treatments. First clinical trials are underway. The randomized phase II/III FEATHER clinical trial at the Paul Scherrer Institute in collaboration with the University of Zurich Animal Hospital is one of the first curative domestic animal trials to be attempted, and it is designed to provide a good example for human trials. However, the lack of standardized quality assurance (QA) guidelines for FLASH clinical trials presents a significant challenge in trial design.

**Purpose:**

This work aims to demonstrate the development and testing of QA and reporting procedures implemented in the FEATHER clinical trial.

**Methods:**

We have expanded the clinical QA program to include UHDR‐specific QA and additional patient‐specific QA. Furthermore, we have modified the monitor readout to enable time‐resolved measurements, allowing delivery log files to be used for dose and dose rate recalculations. Finally, we developed a reporting strategy encompassing relevant parameters for retrospective studies.

**Results:**

We evaluated our QA and reporting procedures with simulated treatments. This testing confirmed that our QA procedures effectively ensure the correct and safe delivery of the planned dose (3%/3 mm gamma criteria, pass *>* 90%). Additionally, we demonstrated that we could reconstruct the delivered dose and dose rate using the delivery log files.

**Conclusion:**

We developed and used in practice a comprehensive QA and reporting protocol for a FLASH clinical trial at the Paul Scherrer Institute. This work aims to establish guidelines and standardize reporting practices for future advancements in the FLASH‐RT field.

## 1 INTRODUCTION

Over the last 10 years, significant research has focused on the FLASH effect, which demonstrates that at ultra‐high dose rates (UHDRs), normal tissue is spared while providing equivalent tumor control compared to conventional (CONV) radiotherapy.^1^ Studies have explored both in vitro and in vivo models to understand the underlying mechanisms and to identify the parameters and conditions necessary to achieve this effect. However, the lack of standardization in experimental reporting and procedure makes it challenging to cross‐compare experiments and reproduce results.^2^, ^3^ Comprehensive reporting to retrospectively determine optimized FLASH delivery parameters, including dose and dose rate,^4^ is critical for successful translation from preclinical studies to clinical applications. Accurate reporting is only meaningful when supported by quality assurance (QA) procedures and robust dosimetry that ensure reliable and reproducible doses and dose rate measurements. This is even more important in the context of first clinical trials.

As first trials of UHDR treatments have started,^5^, ^6^, ^7^, ^8^, ^9^, ^10^, ^11^ researchers and scientific organizations in the field of radiation oncology have discussed how to define QA goals and requirements for UHDR.^12^ A major challenge is that a precise definition of the physics parameters determining the FLASH effect is currently unknown, and consequently, the requirements for QA are also unclear. An initial framework for QA and reporting in UHDR clinical trials has been proposed^13^ highlighting technology gaps and limitations that need to be addressed for the safe implementation of UHDR radiation treatments. Although practical guidelines for machine QA have been developed^14^ and recommendations outlining minimal and optimal requirements have been published,^15^ further validation is necessary. To the best of our knowledge, no consensus has been reached yet.

In this work, we aim to present the development and key considerations of QA processes and reporting procedures for the randomized phase II/III FEATHER clinical trial conducted at the Paul Scherrer Institute in collaboration with the University of Zurich Animal Hospital. The procedures were developed in 2023, and the trial was opened for recruitment in 2024. As most of the studies mentioned above were not yet published at the time of development, we had to look for pragmatic but safe solutions to this problem. In reporting our experience, we want to provide the community with a practical example of reporting and QA procedures, which may serve as a model for future clinical and pre‐clinical studies.

## 2 METHODS

In this section, we will introduce the context of this work, namely the FEATHER trial, which has never been presented before. Further, we will address our framework for QA^13^ in particular, how we are ensuring that the treatment delivery is (1) safe, (2) as planned, and (3) reproducible.

### 2.1 The FEATHER trial

The FEATHER trial (FEline orAl squamous cell carcinoma to model human Head&Neck tumors: A phase II/III randomized trial assessing early toxicity and anti‐tumor efficacy of UHDR vs. conventional dose rate proton THERapy) is a curative trial investigating the FLASH effect in feline biopsy‐confirmed oral squamous cell carcinoma, jointly run by the University Animal Hospital, Zurich, and the Paul Scherrer Institute.^1^ Feline oral squamous cell carcinoma (oSCC) is a naturally occurring and locally aggressive disease that is resistant to treatment. It shares significant pathological and clinical characteristics with human oSCC.^16^, ^17^, ^18^ Median survival times rarely exceed 1 month^19^, ^20^ and current radiation protocols are limited due to toxicity and the tumor's inherent radio‐resistance. Consequently, oSCC serves as a valuable model for investigating the FLASH effect.^21^

Patients are randomized in a CONV and UHDR arm of proton therapy delivery. To avoid bias related to any assumption on the FLASH effect and its “threshold dose or dose rate,” only the beam current varies between the two arms; the CONV arm is defined by a proton beam current below 1 nA (0.76 nA at the patient position), and the UHDR arm by the current that maximizes dose rate (382 nA at the patient position). For reporting purposes, we define the dose rate according to the Folkerts PBS‐average dose rate definition.^22^ In the conditions above, the dose rate is estimated to be, on average, 0.4 Gy/s for the CONV arm and above 50 Gy/s in the UHDR arm for the expected tumor size (*<* 10 cm^3^). Treatment planning and field design are the same in both arms of the study, with no optimization constraints on the dose rate. Each patient receives three fractions of 11 Gy (physical dose) delivered over three consecutive days, amounting to a total of 33 Gy. The treatment regimen was chosen, as it has been seen to produce the FLASH effect^23^ and its BED and EQD2 are comparable to the 10×4.8 Gy regime reported in the literature.^17^ Additionally, as the cats require a catheter change every 3 days, this fractionation scheme simplifies management and reduces the need for extended hospitalization. Further, treatments are scheduled from Friday to Sunday to allow for flexibility, as there are no human patients during that time. The treatment utilizes PSI Gantry 1 with transmission proton beams (250 MeV) and pencil beam scanning without field shape modifying devices. All treatment plans incorporate three different fields, each with equally weighted spots laterally shaped to cover the target. Beam direction and couch position are manually optimized to ensure a uniform dose to the target area and meet dosimetric requirements and constraints for organs at risk. Each treatment session involves beams from only one angle to minimize the effects of “split dose.”^24^

The animals’ follow‐up takes place at the University Animal Hospital in Zurich, but the arm allocation is unknown to the veterinary radiation oncologists to avoid bias. We examine two key endpoints: the primary is acute toxicity affecting mucous membranes, skin, and other vulnerable organs, such as the eye, and the second is tumor control.

### 2.2 Framework for quality assurance

#### 2.2.1 Safety and dose delivery control

In clinical trials involving UHDR irradiation, we align safety objectives with those established for standard clinical operations, as recommended by recent literature^12^, ^13^, ^15^.

**FIGURE 1:**
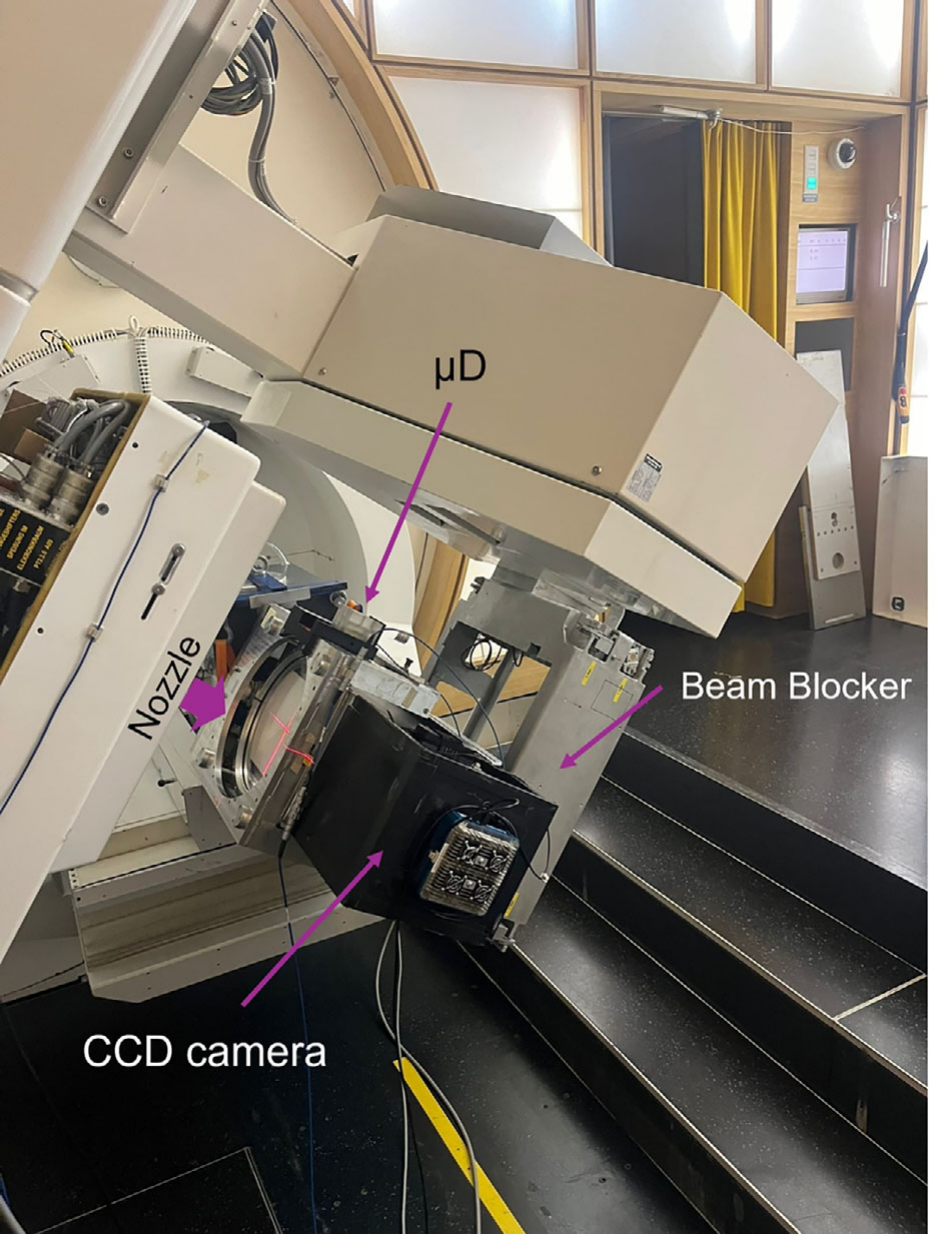
In‐house QA phantom designed for the QA procedure: CCD mounted to a rotation stage.

For the FEATHER trial, we leverage the clinical infrastructure of PSI Gantry 1,^25^ which successfully treated human patients from 1996 to 2018. We have modified this facility into a UHDR beamline^26^ incorporating essential redundancy and safety measures. Additionally, we have replaced the original proton radiography panel^27^ with a beam blocker made of PMMA and copper. Figure 1 shows where the beam blocker is placed and how it rotates with the gantry to stop the beam, thereby preventing secondary neutron production in the ceiling. However, the high beam currents associated with UHDR delivery introduce unique challenges, particularly the potential for significant dose errors resulting from interlock failures.

The Gantry 1 interlock chain exhibits sufficiently rapid reaction times, allowing us to categorize, following the AAPM TG100^28^ and IEC^29^ guidelines, the most probable sources of interlock failure as type‐B hazards or lower. As a result, we have not altered the interlock chain. Most of the limits for interlocks are based on dose measurements and, therefore, have not been modified for the trial. We modified only the maximum spot duration check, now defined separately for CONV and UHDR deliveries. This careful approach guarantees safe irradiation delivery for both modalities in compliance with established standards^29^ and based on more than 20 years of clinical operation.

It is crucial to note that interlocks and subsequent beam delivery interruptions during dose application can influence the delivery time and, therefore, the dose rate. Therefore, any interlocks are documented, and if an interlock happens during a UHDR treatment, the patient is excluded from that arm (even though the patient will be followed up as planned). However, we expect only a few such occurrences (and, in fact, there has been no interlock interrupting delivery so far).

Regarding dose delivery control in the FEATHER trial, we allow beam currents at the patient location to reach a maximum of 400 nA. We found recombination effects in the dose control monitor chamber (the gantry nozzle ionization chamber) under UHDR up to 20% at 600 nA.^26^, ^30^ While we must account for these effects, their magnitude does not preclude the use of an ionization chamber for beam current control. Based on our operational experience with Gantry 1 as a UHDR beamline, we observe that charge density and transmission are stable during a full day of operation, although daily variations occur.^26^ Therefore, during our QA procedure for UHDR treatment, we calibrate the dose control monitor chamber daily to ensure dose delivery accuracy.

#### 2.2.2 Quality assurance

In the FEATHER trial, we optimize patient treatment plans exclusively based on the administered dose, disregarding the dose rate. Consequently, from a QA perspective, the primary focus is on ensuring the accuracy of the delivered volumetric dose, which remains invariant across both treatment arms. We adhere to established QA guidelines for proton therapy using the existing QA procedures from Gantry 1. Since we use a single energy, however, we have reduced the number of QA tests compared to the previous clinical program.

As we expect the patient frequency to be approximately 1/month, we separated the QA testing into:

- Yearly tests;
- Treatment‐week and patient‐specific (PSQA) tests, to be performed within 1 week of a new treatment as part of the verification procedure of a new starting patient;
- Daily tests (DQA) are to be performed only on treatment and verification days.

While the PSQA aims to verify that the correct dose is administered at the intended location, the DQA confirms that the delivery on that specific day aligns with the planned and verified delivery.

Machine performance and beam model consistency will be evaluated over time as part of the annual QA protocol following standard clinical practices. During the week of patient treatment, we perform checks on the delivery chain, such as verifying the reproducibility of the dose measured by the dose control monitor chamber.

##### Patient‐specific QA

For patient‐specific QA, we designed a measurement device encompassing a CCD camera based on a scintillating foil and a micro diamond detector (*µD*).^31^ All detectors have been verified to be dose rate independent.^32^ The CCD camera and the *µD* are dose cross‐calibrated to our institute's reference chamber, traceable to a primary standard. The measurement is performed at a depth of 2 cm, which aligns with the expected depth for most tumors in the trial. Further, using a 250 MeV transmission field, we do not expect measurement depth to affect the field shape and uniformity. The device can rotate so that the measurement plane is always perpendicular to the gantry angle. Figure 1 presents a picture of the device. The *µD* can be connected to the gantry front‐end electronics and measures the current induced by impinging proton radiation. Thanks to the 1 kHz sampled readout of the current, the *µD* can be used for dose rate calculation.

The patient‐specific QA consists of the following steps (for each patient field):

- Test of the alignment of the beam isocenter with the cross‐hair lasers, which are used for patient positioning, given that our gantry on‐board imaging system is out of operation.

The tolerance is set to 2 mm.

- Evaluation of the spot position and spot size: a 5‐spot pattern is delivered, measured with the CCD camera, and compared with the commissioning data. The allowed deviation for positioning is 2 mm, and for beam size (fitted with a 2D‐Gaussian) ± 10%. If necessary, a position offset is calculated to correct for any systematic positional shifts.
- Test of the delivered dose, measured with the *µD*. The measurement is performed only at the center of the field as the fields are uniform. The measured and planned dose ratio should be within 5%. If not, the field dose is scaled accordingly. In UHDR mode, we determine the dose control monitor recombination factor through our recombination‐free micro diamond reference detector.
- Test of the patient field delivery: after eventually applying the corrections defined above, the 2D dose distribution is measured with the CCD. The dose difference between the delivered and the planned dose in the 90% isodose area is calculated. If the average deviation is larger than 3%, a boosting factor is determined, and the delivery is repeated. To evaluate differences in the spatial dose distribution, we perform a gamma analysis with distance and dose threshold levels of 3 mm/3%. The pass criterion is met when more than 90% of the voxels within the 90% isodose area are at *γ* ≤ 1. The smooth gamma criterion accounts for the approximation made by the treatment planning system (TPS), which assumes an average beam size within the spot map for each angle, despite the actual beam size exhibiting variations of up to 10%. If the agreement is still unsatisfactory, we make a clinical decision based on the location of the largest deviation.

In addition, we measure the local dose rate at different positions within the field using the *µD* to ensure that it is above 40 Gy/s^1^ in the UHDR case. If this requirement is not met, even though such a situation is unlikely based on our experience, we will adjust the spot positioning in the treatment plan by reducing the number of spots.

##### Daily QA

The DQA procedure is oriented toward evaluating machine conditions and safety checks on the day of the treatment. The dose and especially the dose rate map of the field are very sensitive to phase space and beam position variations; therefore, these parameters are evaluated on a daily basis and compared to the PSQA conditions as recommended by current best practice.^12^, ^15^ From experience,^26^ we know that these parameters are stable within a day of operation. Therefore, we check them only before treatment as part of the DQA procedure. The DQA also includes machine interlock tests to ensure the system responds correctly.

The DQA procedure is based on the previously described PSQA, but the reference dose distribution is not the recalculated dose in water from the TPS, but the dose delivered on the day of the PSQA. The tolerances used are the same as the ones presented for the PSQA.

Additionally, in the case of UHDR delivery, we correct for recombination effects.

#### 2.2.3 Data recording

We modified the recording concept used for monitoring in Gantry 1 during patient treatments^25^ by establishing a time‐resolved data recording system. This setup enables us to generate time traces for reconstructing the delivered dose in a retrospective and time‐dependent manner, enabling a post‐delivery reconstruction of the dose distribution and dose rate map. Therefore, the data are recorded with a sampling frequency of 1 kHz. The resulting log files contain the following information (additional information is available in Table A1):

- Delivered dose as a function of time, recorded by two independent dose monitors; • Hall sensor values for two Hall probes positioned in the scanning magnets;
- Time stamps at 100 *µ*s resolution.

Continuous sampling starts as soon as the patient field is loaded for delivery, independently of dose delivery and beam application. This allows for the evaluation of the machine's behavior during beam pauses, including the magnets’ ramping and settling time.

We derive the scanning magnet current from Hall sensor readings using a calibrated curve. Subsequently, we translate these magnet currents into lateral spot position coordinates using commissioning data.

We describe each irradiation spot as a two‐dimensional Gaussian distribution. The mean positions *µ* are calculated from the Hall sensor values converted into corresponding spot coordinates. The beam widths *σ* are extracted from commissioning data, while the integral dose is determined from the logged dose values corresponding to dose monitor counts. By summing all individual Gaussian distributions, we generate a 2D dose map of the irradiated field.

Furthermore, by incorporating the time stamps *t_i_* at the termination of each spot, we can track the temporal evolution of the 2D dose map. We utilize Folkert's metric^22^ to then compute the PBS‐average dose rate at 5%–95% dose levels. This method enables us to calculate the dose rate map for the delivered field across all voxels.

#### 2.2.4 Reporting

As the final report of the treatment course, we developed a comprehensive protocol to report information relative to the patient, the machine characteristics, the treatment delivery, the plan evaluation, and relevant information from the QA test, such as the dose, the dose uniformity, and the dose rate. The included information is reported in Table 1.

**TABLE 1:** Summary of the information included in the report.

Category	Description
Patient‐specific parameters	Veterinarians collect patient‐ and tumor‐specific characteristics, including age, breed, sex, histology, and tumor staging.
Machine‐specific characteristics	Reported parameters include: Beam energy and structure (pulse width, frequency) Cyclotron and post‐nozzle currents (considering beam transmission)
Setup	Reported parameters include: Patient positioning, couch and gantry angles for each field Anesthetic and patient parameters (e.g., blood oxygen saturation)—recorded before and during irradiation with a pulse oximeter and capnograph.
Dosimetric characteristics	The treatment plan is exported as DICOM. Key dosimetric metrics include: DVHs, DMin, DMean, DMax, V95, D98, and D5‐D95 Dose distribution screenshots Patient‐specific QA: recalculation in water, gamma analysis, and log file comparisons
Dose rate (not shared before unblinding)	Reported data include: Dose‐averaged^39^ and PBS‐average dose rates^22^ calculated in the TPS Irradiation time per field PBS‐average dose rate measured with *µD* detector Log files for dose and dose rate reconstruction (as a separate file)

## 3 RESULTS

### 3.1 Quality assurance

**FIGURE 2:**
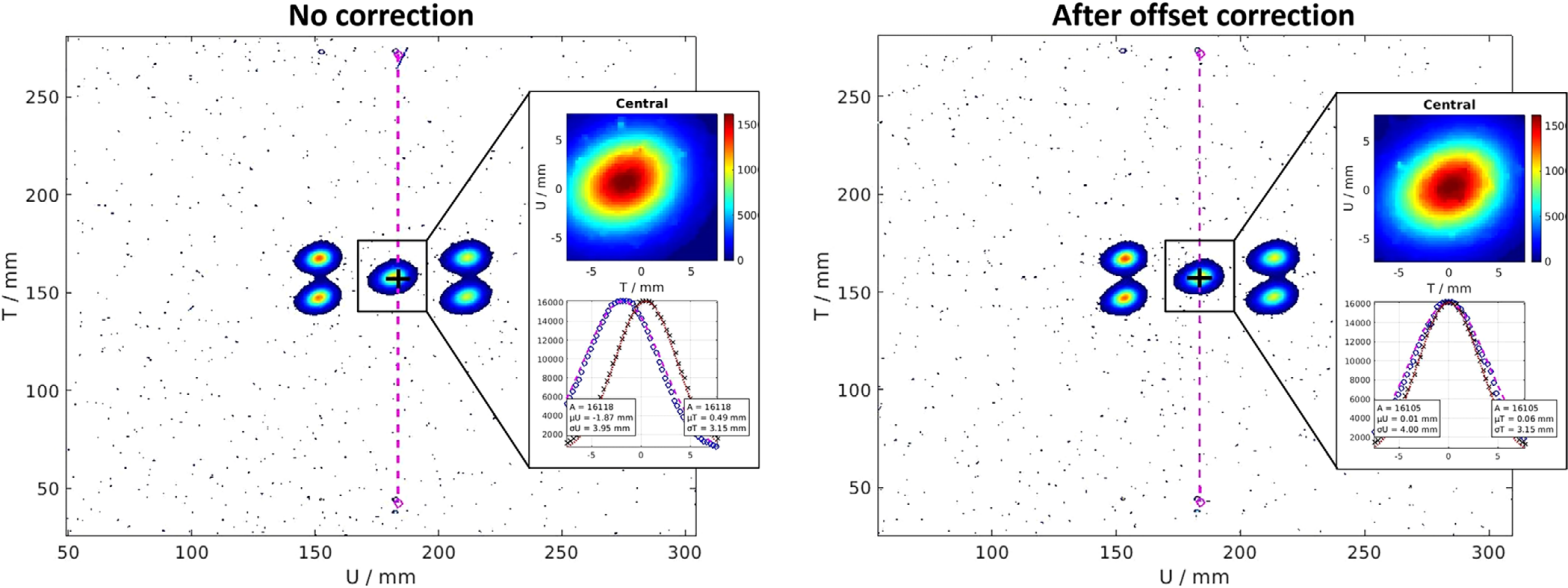
The five‐spot pattern is delivered to evaluate spot positioning and beam size (left). The purple line represents the reference central axis that passes through the center of the cross‐hair laser. The first delivery is used to evaluate the offset. After calculating the offset, a second delivery with the corrected spot position is performed to confirm the results (right).

Figure 2 shows results from the first step of our PSQA and DQA tests: the beam position check. We center the CCD camera mounted on the QA phantom with the cross‐hair lasers and deliver the simple five‐spot pattern. The central axis is marked on the CCD and is visible during the analysis, serving as a reference for calculating the beam offsets. Figure 2 illustrates the five‐spot pattern before and after the offset correction, with the central spot now centered relative to the cross‐hair lasers.

**FIGURE 3:**
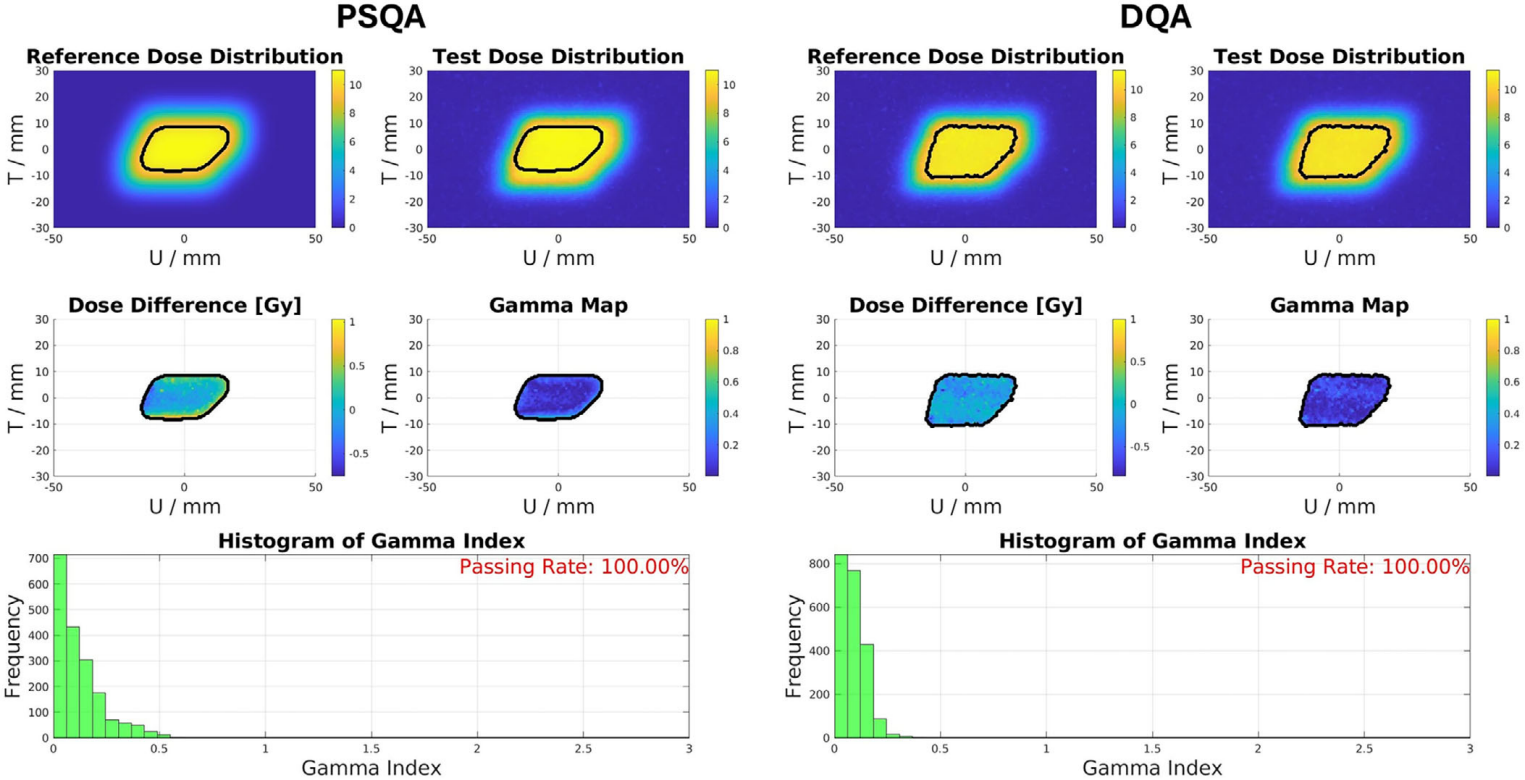
2D dose distribution of the reference and test fields, including the dose difference and gamma analysis between the two, along with an evaluation of the gamma index. On the left, we display the PSQA analysis, where the reference represents the dose distribution recalculated in water from the TPS and the test dose distribution, the measured field. On the right, the reference field is the dose delivered on the day of the PSQA. (left) *µD* detector current readout during a UHDR beam delivery. (right) The cumulative sum of the dose delivered.

Figure 3 shows the measurements of the 2D dose distributions [in the U (horizontal) T (vertical) plane] performed in the PSQA and in the DQA. The 2D dose distribution measured with the CCD camera is compared to the 2D dose distribution recalculated (using the ray‐casting dose calculation algorithm^33^) by the TPS in water for the PSQA, or to the measured PSQA dose distribution for the DQA, using average dose difference and gamma analysis for doses above the 90% isodose level.

For the presented case in the PSQA [Figure 3 (left)], we found a high level of agreement within the 90% isodose curve. This agreement is expected since variations between the beam model imported into our TPS and the actual beam characteristics are more pronounced at the edges of the spot map and at gantry angles close to 0°, as the beam transport was optimized^26^ for a fixed gantry angle of 90° for the central spot. The TPS cannot accurately simulate these variations, as the beam model remains invariant within the spot map of each scanning angle.

In the DQA just before treatment, the validated 2D dose distribution from the PSQA is ultimately compared with the daily measurement. Figure 3 (right) shows the analysis for this case. Instabilities in the beam's position or size, even those small enough to pass spot position and size checks, may cause visible deformations.

**FIGURE 4:**
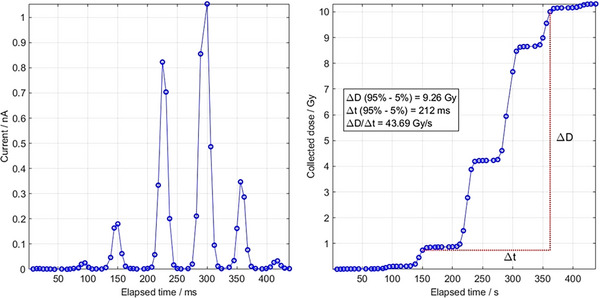
Dose map recalculated from log files (left), measured with the CCD camera (center), and calculated by our TPS (right). The dose measured by the *µ*D detector in the reference point is 10.3 Gy.

In addition to dose verification, our workflow includes dose rate measurement at different positions within the field. Figure 4 (left) illustrates an example of *µD* data sampled during a UHDR delivery at a frequency of 1 kHz. To calculate the dose from the measured *µD* output current, we first convert the current to accumulated charge and then to dose using the *µ*D calibration factor. The time resolution of the readout is sufficient to compute the PBS‐average dose rate, as shown in Figure 4 (right).

### 3.2 Dose and dose rate reconstruction from log files

**FIGURE 5:**
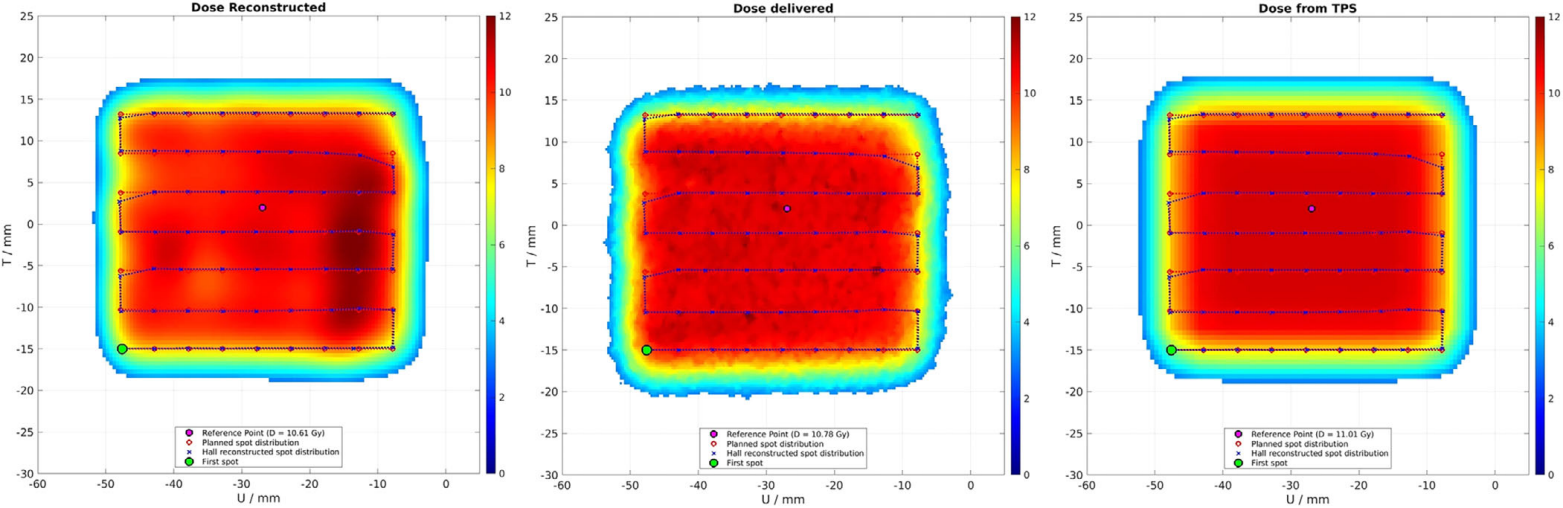
Dose rate map recalculated from log files (left), and calculated by our TPS (right). The dose rate measured by the *µD* detector in the reference point is 54.3 Gy/s. Additional information are in the Appendix (Figure A1 and A2).

In Figure 5, we compare, for an example field, the dose reconstructed from the log files with the dose measured with the CCD camera. For reference, we also plot the dose recalculated in water from the TPS. Further, we report the reconstructed, the measured (with CCD), and the simulated (with TPS) dose at an arbitrary position within the treatment field, and we compare it with the dose measured with the *µ*D.

It is important to mention that the TPS works with a simplified Gantry 1 beam model. For each gantry angle, we averaged the mean spot size in T and U and the integral (Gy/MU—monitor units), and we assumed these to be constant over the whole map but variable over gantry angles. In addition, we performed dose and dose rate calculations, placing the spots evenly in a rectangular grid. For the dose rate, we assumed a constant spot‐changing time in U (4 ms) and T (11 ms), and constant beam intensity MU/s. This simplifies the calculation, as we actually have a variable beam size and integral across the spot map. These factors may explain the dose and dose rate distribution variations, particularly at the edges of the spot map. Moreover, the TPS uses a default cutoff factor for the beam spread to prevent excessive computational demands. For the log file recalculation, each spot distribution, represented as a 2D Gaussian, is calculated and then summed across the entire calculation grid.

In Figure 5, we observe that the difference between the reconstructed dose and the delivered dose at the reference position is smaller than 2%, and the measured field shape aligns well with the reconstructed data, indicating good agreement with the reconstructed 2D Gaussian distributions. The calculation from the log files overestimates the dose on the right side of the field. This could be explained by the spot position adjustment in the UHDR delivery required to reduce delivery time.

**FIGURE 6:**
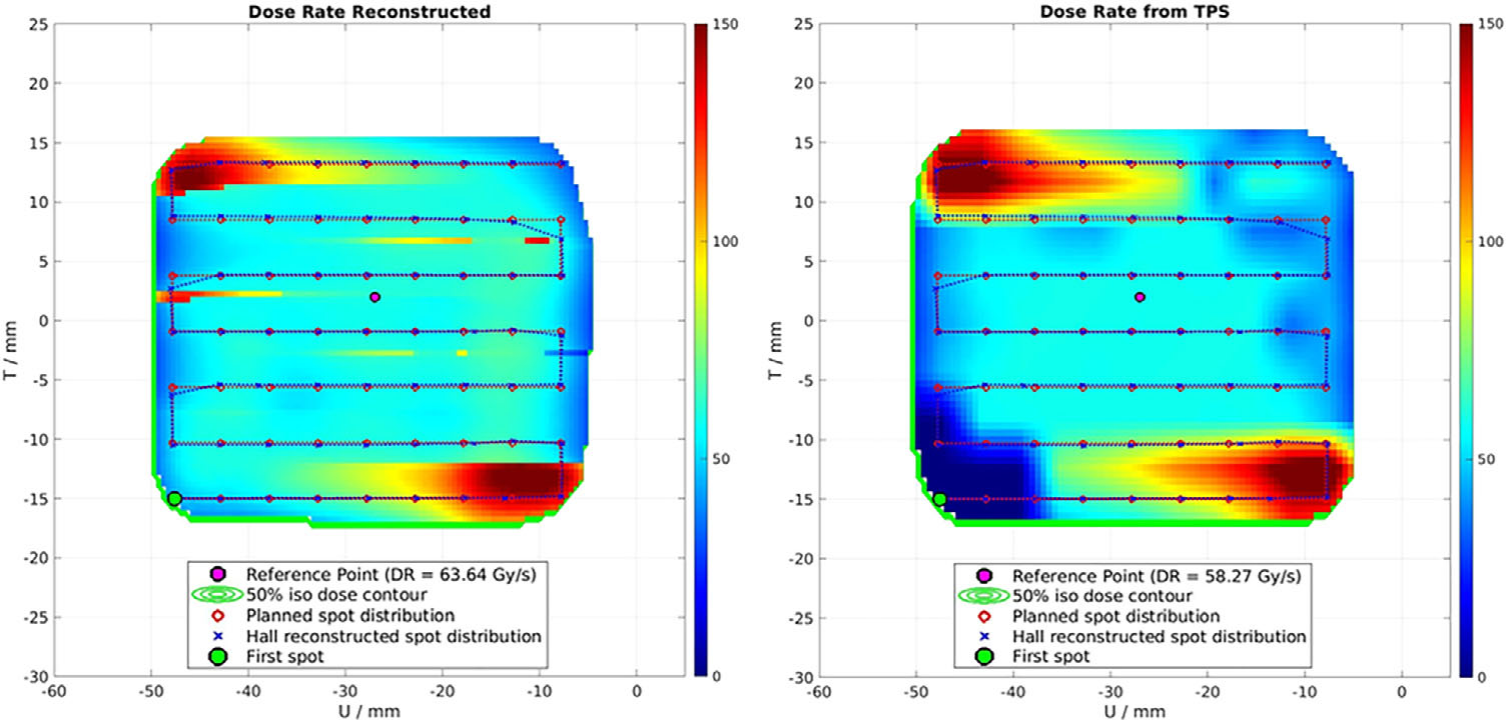
Screenshot of the delivery report for an example patient.

In Figure 6, we compare the dose rate reconstructed from the log files with the one simulated by our TPS. At the reference position, the difference in dose rate is within 10%, which is acceptable given the uncertainties in the delivery and the differences between the log file and dose recalculation. In the reconstructed dose rate, we observe very sharp variations between neighboring points, particularly at the edges of the field. This is due to the method used to calculate the PBS‐average dose rate. The lower or upper threshold (5% and 95% of the maximum dose, respectively) in the cumulative dose graph can fall just before or after the plateau, which corresponds to the time when the beam is off and the scanning magnets are changing current for the next spot, as shown in the Appendix (Figure A1). The position of the starting and endpoint of the calculation impacts the dose rate calculation. This behavior is not observed in the TPS dose rate calculation, as a step‐like function approximates the cumulative dose. In this model, the dose per spot is constant while the beam is on, as illustrated in the Appendix (Figure A1 and A2). Although this is a simplified model, it provides a good approximation for our needs since the delivery (in 1 kHz sampling rate) is almost instantaneous.

### 3.3 Reporting

Given the trial's blinded design, the delivery report is divided into three parts, with only the first one made available to the veterinary radiation oncologists before unblinding. The first part includes the dosimetric evaluation of the plan, detailing the 3D dose distribution, prescription evaluation, and dose‐volume histograms. The second part consists of dose rate considerations, including measurements with the micro diamond and TPS calculations (dose distribution and dose rate volume histograms). The final part is reserved for the PSQA and DQA.

**FIGURE 7:**
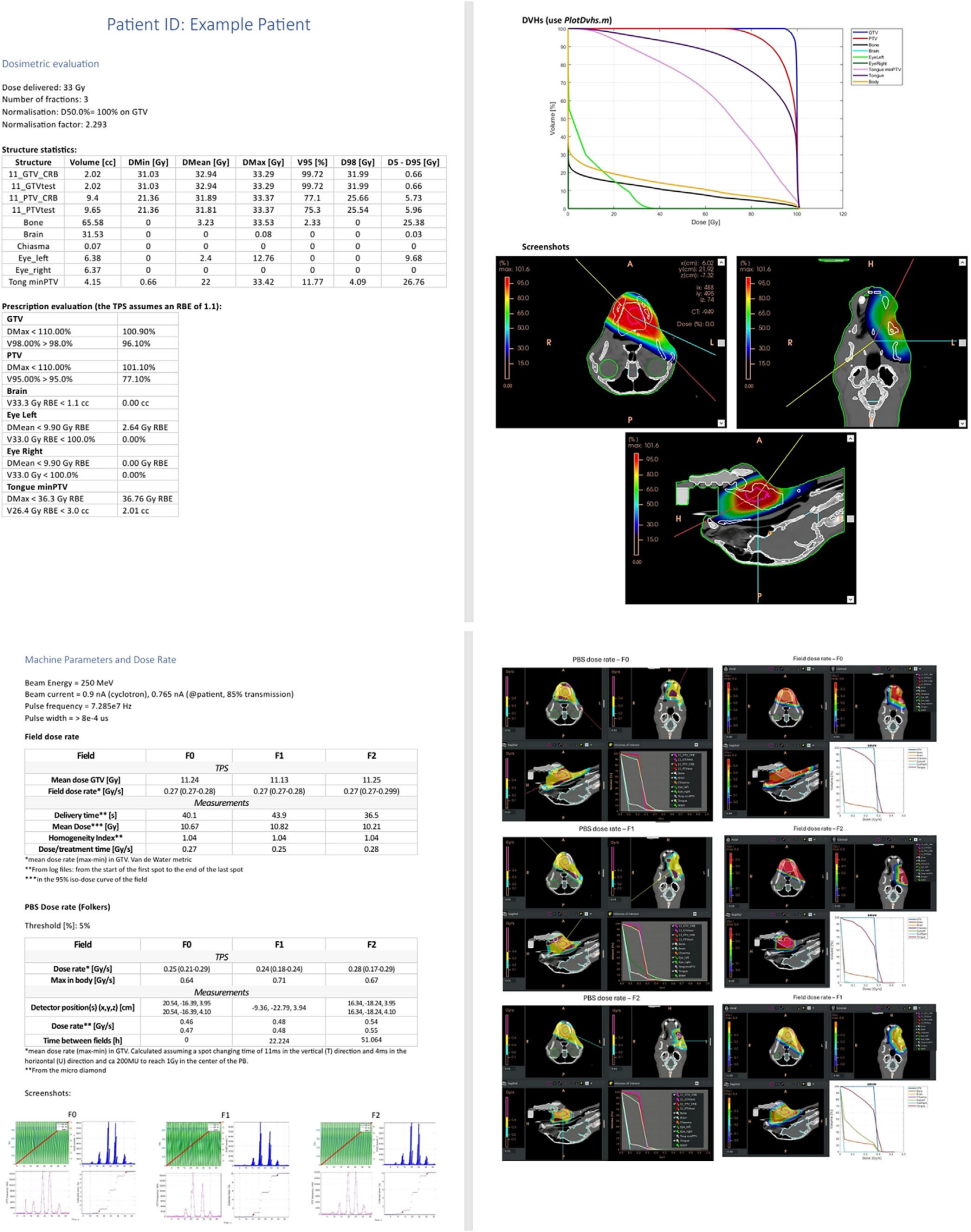
Screenshot of the delivery report for an example patient.

For completeness, we provide the additional materials with a delivery report of a test patient; a preview is shown in Figure 7.

Given the blinded nature of the study, information about dose rate and beam current will be stored in our institutional database and can be disclosed only at the end of the study for each patient.

## 4 DISCUSSION

This work focuses on two separate aspects of designing a FLASH clinical trial. The first is the QA process, which contains some specific UHDR tests, such as recombination, timing, and interlocking the beam in UHDR, which requires different time limits for safe dose delivery, and phase space checks due to slight differences in phase space between UHDR and CONV irradiation. This QA strategy is currently being used in the FEATHER trial. The second aspect involves reporting. Due to the absence of specific guidelines during the development of our protocol and considering this is the first proton PBS curative trial, we need to document and report more information than is typically required for feasibility and Phase I trials.^5^ To address this, we have created a comprehensive protocol that includes all necessary parameters for retrospective analysis and recalculation. To the best of our knowledge, this article provides the first QA and reporting protocol specifically designed for and applied in a proton FLASH phase II/III clinical trial.

The main goal of moving preclinical research into clinical trials of UHDR radiotherapy is to evaluate the FLASH effect and assess the feasibility and reproducibility of delivering UHDR treatments. When defining a FLASH trial, however, the first challenge is adapting safety and QA best practices at low to high dose rates. Safety recommendations currently in use,^29^ however, are not adequate for UHDR. In addition, a FLASH trial requires reporting the time behavior of the dose distribution, which in the past was irrelevant in RT. Work is currently ongoing in international working groups, addressing these open questions^12^, ^13^, ^14^, ^15^.

In the FEATHER trial, we are not specifically optimizing for dose rate (although the dose rate in the field is verified to be above 40 Gy/s^1^), as the definition of dose rate varies across studies and delivery modalities; our focus is solely on the dose, and our treatment plans remain the same for both the CONV and UHDR arm. Therefore, as part of our clinical trial's QA process, we focused primarily on ensuring the accuracy and precision of the dose delivered to the patient. Consequently, we can refer to the established QA guidelines for proton therapy. A key advantage of our approach is that it relies on procedures and instruments already used in clinical practice without requiring extensive adaptation or new developments. Our in‐house‐built QA phantom can be substituted with any similar clinically used daily QA device. Spot size and field quality measurements can be performed with commercial devices^14^ and various solutions have been proposed for time‐resolved dose measurements.^34^, ^35^, ^36^ In our case, we adapt the readout of our instrumentation to provide a time‐resolved dose measurement and allow for log file calculation of time traces, as proposed in the literature.^37^ This permits us to recalculate parameters such as dose rate if a new definition becomes relevant.

With this pragmatic approach, we have demonstrated that many existing clinical procedures can be successfully adapted to meet the requirements of early UHDR clinical trials, where dose rate optimization is either minimal or absent. We believe our approach can apply to upcoming studies since the preclinical knowledge of the FLASH effect does not yet allow its full exploitation. Trials in the next years will mainly focus on increasing the beam current and lowering the beam delivery time. In this context, a critical priority should be adapting the monitoring system to allow time‐resolved measurements for recording. Given the short irradiation time, attention should be paid to potential errors due to high beam currents. The risk of interlocks occurring during a delivery cannot be entirely eliminated. A better understanding of how beam pauses influence the FLASH effect would be beneficial in understanding how to proceed in the case of interlocks.

The usefulness of data collected in clinical trials can only be guaranteed by a complete recording and consistent and high‐quality reporting. For FLASH deliveries, where the underlying physics and biology parameters are still under intense investigation, completeness of recording is even more important, as it guarantees that the data can be further analyzed and interpreted as preclinical knowledge progresses. Despite the initial lack of guidelines, our protocol aligns closely with recommendations that were published later.^2^, ^38^ Time‐resolved readouts at very short timescales are crucial to this goal. Dose delivery time traces will be a fundamental part of every future UHDR data analysis and characterization. Both vendors and users of UHDR beams should prioritize this aspect.

## 5 CONCLUSION

We designed a QA strategy to ensure the safe and accurate delivery of treatments in both CONV and UHDR modes used in the FEATHER trial. Additionally, we developed a comprehensive protocol that includes all necessary parameters for retrospective analysis and recalculation. This approach will guarantee that our data could be used in future comparisons across clinical trials, supporting the transition of FLASH research into clinical application. We hope that our experience can serve as guidance for upcoming trials, and that will foster discussions and collaborations within the FLASH community.

## CONFLICT OF INTEREST STATEMENT

The authors have no relevant conflicts of interest to disclose.

## Supporting information



Supporting Informaiton

## Data Availability

The data that support the findings of this study are available from the corresponding author upon reasonable request.

## Footnotes

^1^
The protocol and procedures employed in the clinical trial were ethically reviewed andapproved by the Animal Ethics Council of the Canton of Zurich and Aargau, Switzerland (permit numbers: ZH006/2023).
