# Supplementary material for: Quality assurance and reporting for FLASH clinical trials: The experience of the FEATHER trial
Source: Med Phys. 2025 Sep 3;52(9):e18100. doi: 10.1002/mp.18100 (PMC12409223; doi:10.1002/mp.18100)
Supplement: Supplementary file 1 — Supporting Informaiton [file MP-52-0-s001.pdf]

Appendix

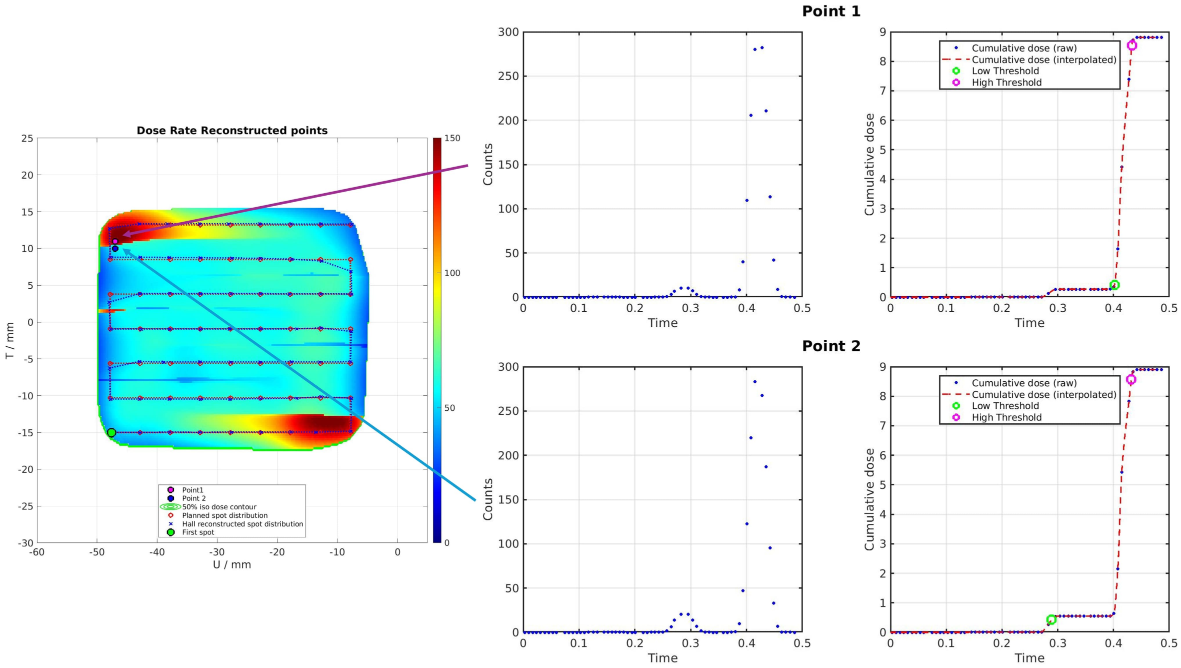

Figure A1: Difference between the dose rate calculation two neighboring points. Even if the count's distribution looks similar as the two points lie close to each other, the position of the lower threshold changes significantly, affecting the dose rate calculation.

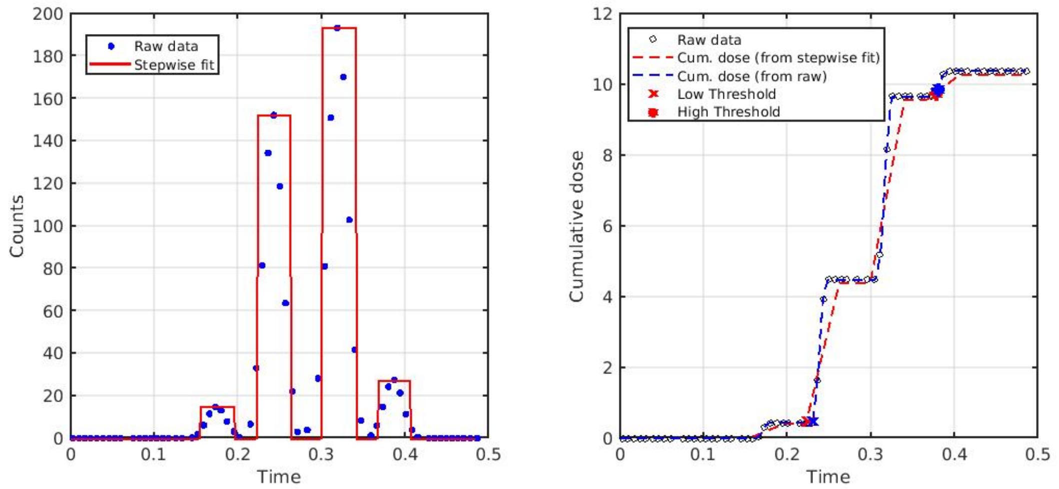

Figure A2: Comparison between log file reconstructed dose rate using a continuous function to represent the counts as a function of time (blue line) and using a stepwise fit of the counts (red line) as an approximation of the TPS calculation.

Patient ID: Example Patient

Dosimetric evaluation

Dose delivered: 33 Gy
Number of fractions: 3
Normalisation: D50.0%= 100% on GTV
Normalisation factor: 2.293

Structure statistics:

Structure	Volume [cc]	DMin [Gy]	DMean [Gy]	DMax [Gy]	V95 [%]	D98 [Gy]	D5 - D95 [Gy]
11_GTV_CRB	2.02	31.03	32.94	33.29	99.72	31.99	0.66
11_GTVtest	2.02	31.03	32.94	33.29	99.72	31.99	0.66
11_PTV_CRB	9.4	21.36	31.89	33.37	77.1	25.66	5.73
11_PTVtest	9.65	21.36	31.81	33.37	75.3	25.54	5.96
Bone	65.58	0	3.23	33.53	2.33	0	25.38
Brain	31.53	0	0	0.08	0	0	0.03
Chiasma	0.07	0	0	0	0	0	0
Eye_left	6.38	0	2.4	12.76	0	0	9.68
Eye_right	6.37	0	0	0	0	0	0
Tong minPTV	4.15	0.66	22	33.42	11.77	4.09	26.76

Prescription evaluation (the TPS assumes an RBE of 1.1):

**GTV**	
DMax < 110.00%	100.90%
V98.00% > 98.0%	96.10%
**PTV**	
DMax < 110.00%	101.10%
V95.00% > 95.0%	77.10%
**Brain**	
V33.3 Gy RBE < 1.1 cc	0.00 cc
**Eye Left**	
DMean < 9.90 Gy RBE	2.64 Gy RBE
V33.0 Gy RBE < 100.0%	0.00%
**Eye Right**	
DMean < 9.90 Gy RBE	0.00 Gy RBE
V33.0 Gy < 100.0%	0.00%
**Tongue minPTV**	
DMax < 36.3 Gy RBE	36.76 Gy RBE
V26.4 Gy RBE < 3.0 cc	2.01 cc

DVHs (use *PlotDvhs.m*)

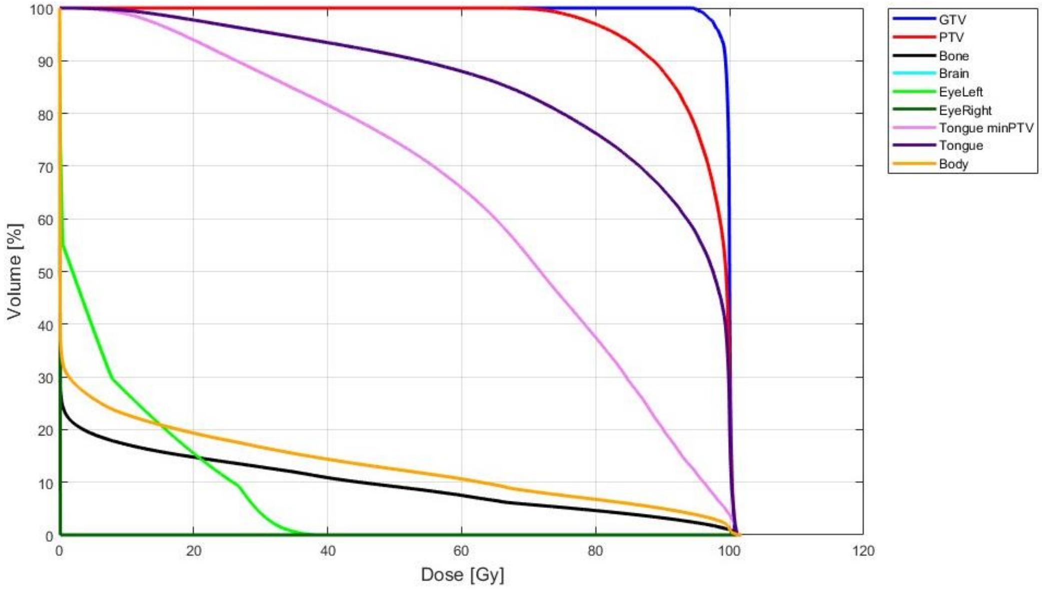

Screenshots

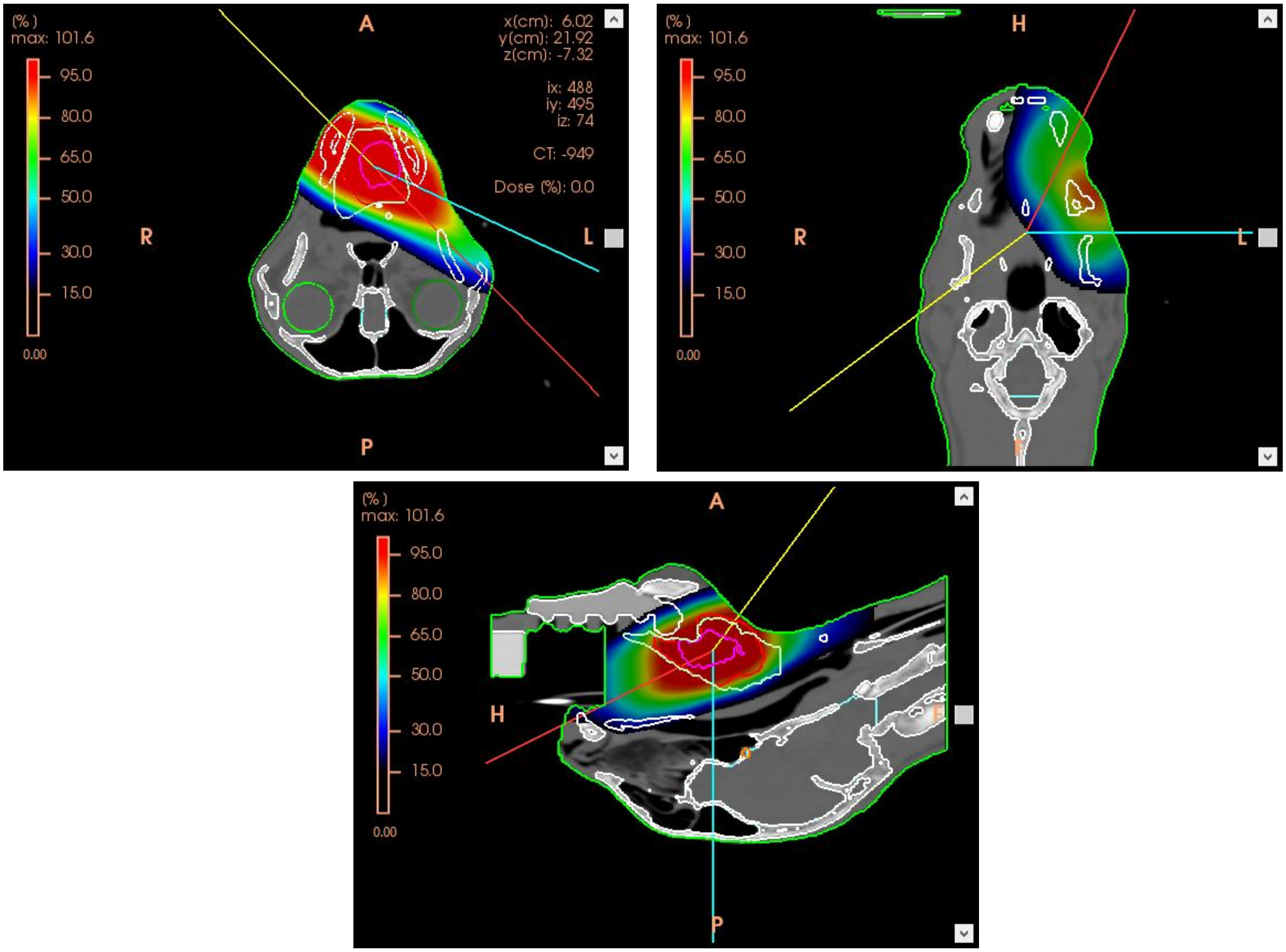

Setup gantry

From TPS:

Field	F0	F1	F2
Field center (x,y,z) [cm]	0.16, 13.64, -7.17	0.16, 13.64, -7.17	0.16, 13.64, -7.17
Alpha (Gantry)	-114	-51	-115
Beta (Table)	-116	37	180
Nozzle [cm]	3	5	5

From CT: Field centre to marker distance

Field	F0	F1	F2
dx	4.46	4.46	4.46
dy	2.26	2.26	2.26
dz	10.88	10.88	10.88

Gantry coordinates:

Field	F0	F1	F2
X [cm]	1.99	-6.86	-3.57
Y [cm]	-4.48	-0.54	-4.16
Z [cm]	29.27	37.94	24.7

Setup patient

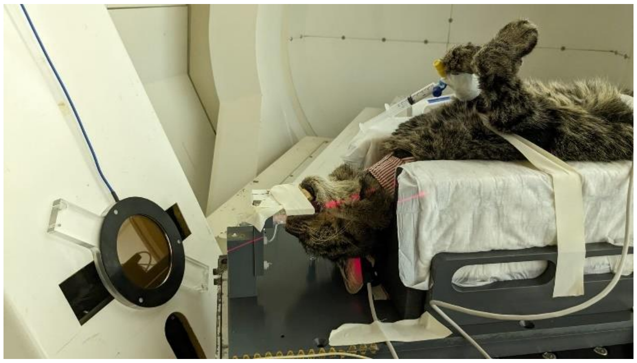

- Beam blocker is required for F0 and F2

Comments and additional Screenshots:

F0

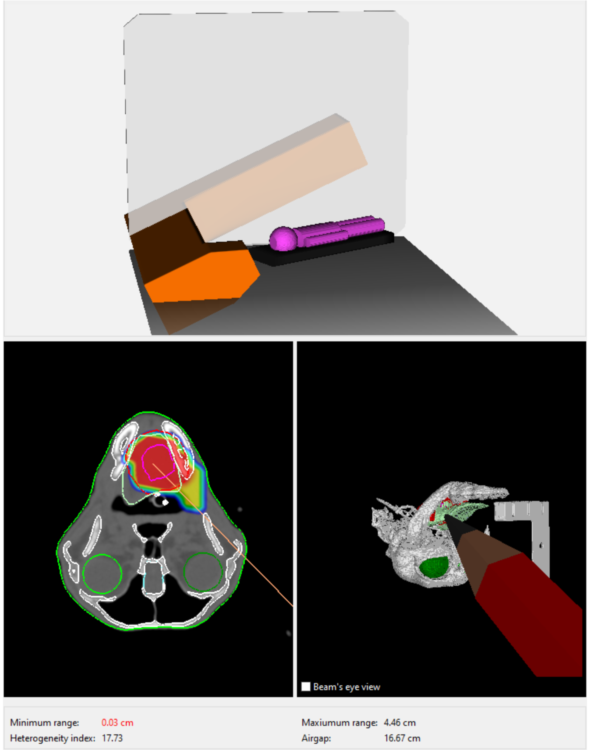

F1

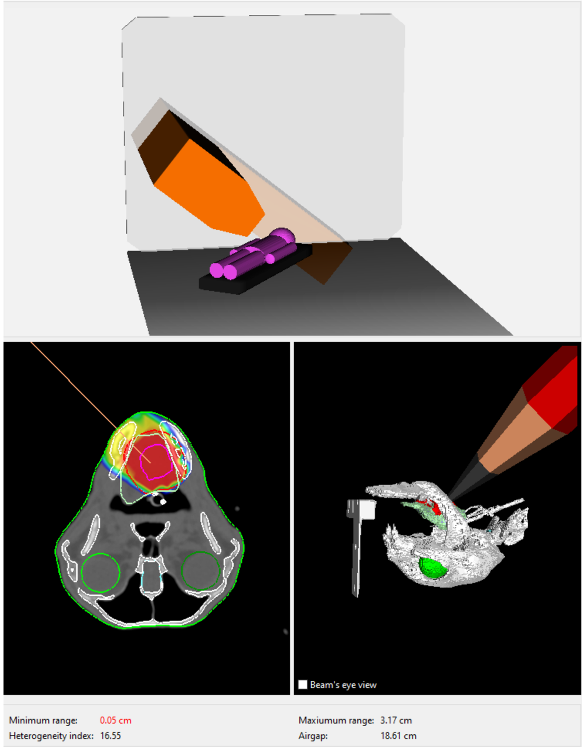

F2

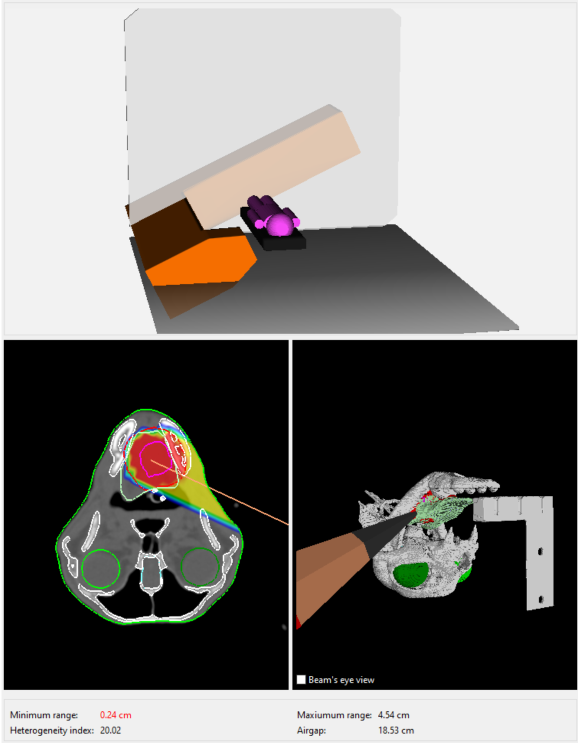

Machine Parameters and Dose Rate

Beam Energy = 250 MeV
Beam current = 0.9 nA (cyclotron), 0.765 nA (@patient, 85% transmission)
Pulse frequency = 7.285e7 Hz
Pulse width = > 8e-4 us

Field dose rate

Field	F0	F1	F2
TPS			
**Mean dose GTV [Gy]**	11.24	11.13	11.25
**Field dose rate* [Gy/s]**	0.27 (0.27-0.28)	0.27 (0.27-0.28)	0.27 (0.27-0.299)
Measurements			
**Delivery time** [s]**	40.1	43.9	36.5
**Mean Dose*** [Gy]**	10.67	10.82	10.21
**Homogeneity Index****	1.04	1.04	1.04
**Dose/treatment time [Gy/s]**	0.27	0.25	0.28

*mean dose rate (max-min) in GTV. Van de Water metric
**From log files: from the start of the first spot to the end of the last spot
***in the 95% iso-dose curve of the field

PBS Dose rate (Folkers)

Threshold [%]: 5%

Field	F0	F1	F2
TPS			
**Dose rate* [Gy/s]**	0.25 (0.21-0.29)	0.24 (0.18-0.24)	0.28 (0.17-0.29)
**Max in body [Gy/s]**	0.64	0.71	0.67
Measurements			
**Detector position(s) (x,y,z) [cm]**	20.54, -16.39, 3.95<br>20.54, -16.39, 4.10	-9.36, -22.79, 3.94	16.34, -18.24, 3.95<br>16.34, -18.24, 4.10
**Dose rate** [Gy/s]**	0.46<br>0.47	0.48<br>0.48	0.54<br>0.55
**Time between fields [h]**	0	22.224	51.064

*mean dose rate (max-min) in GTV. Calculated assuming a spot changing time of 11ms in the vertical (T) direction and 4ms in the horizontal (U) direction and ca 200MU to reach 1Gy in the center of the PB.
**From the micro diamond

Screenshots:

F0

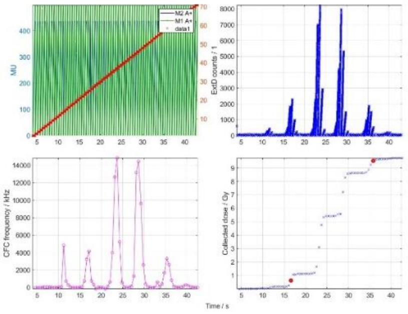

F1

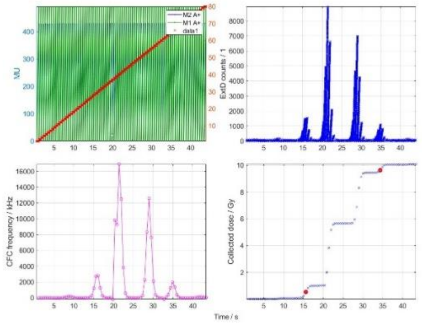

F2

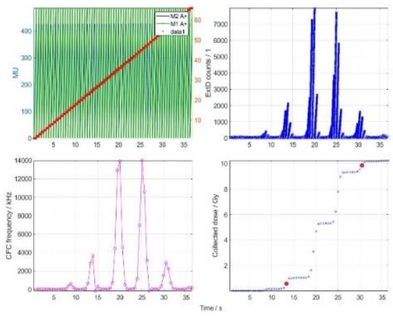

PBS dose rate – F0

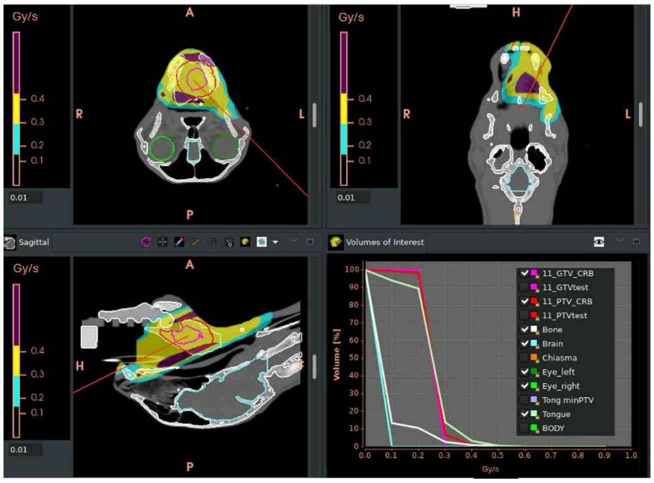

Field dose rate – F0

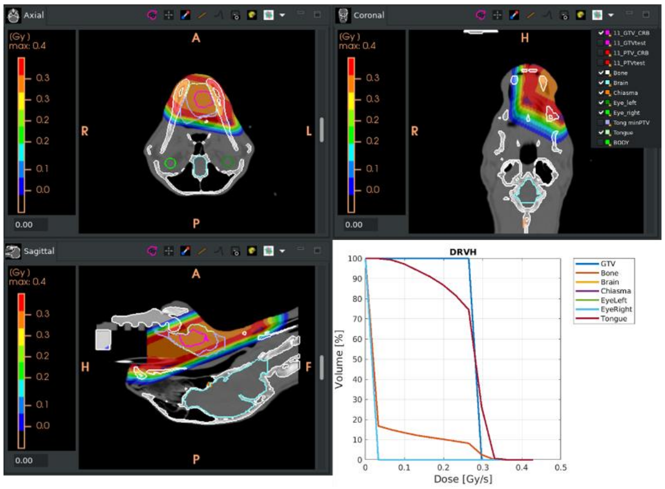

PBS dose rate – F1

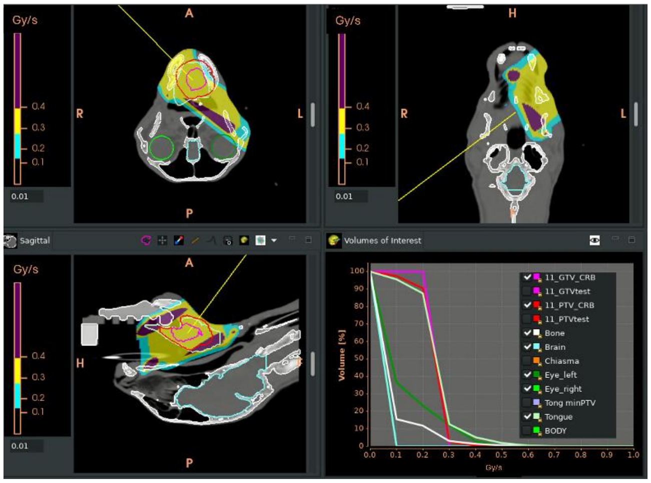

Field dose rate – F2

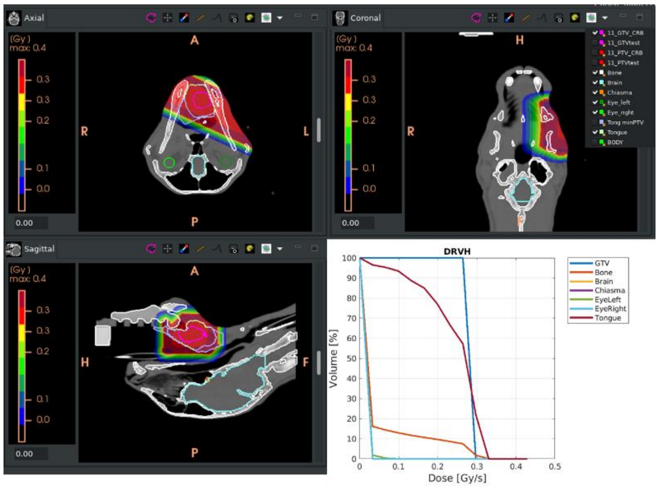

PBS dose rate – F2

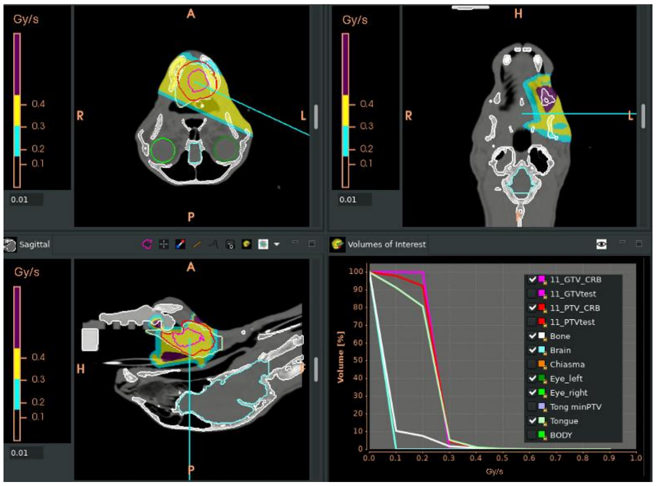

Field dose rate – F1

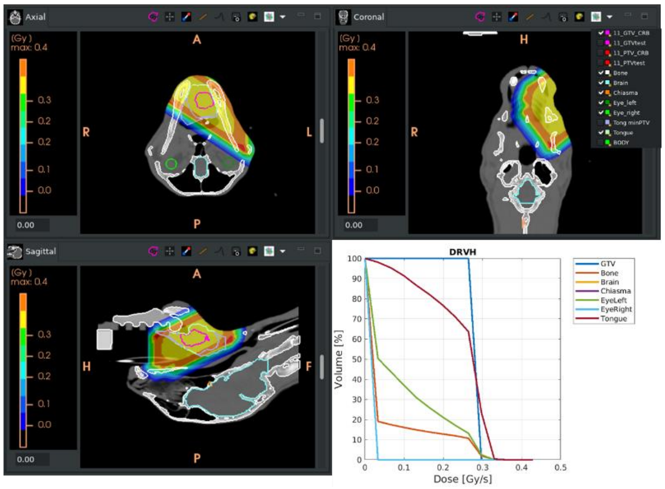

PSQA

F0

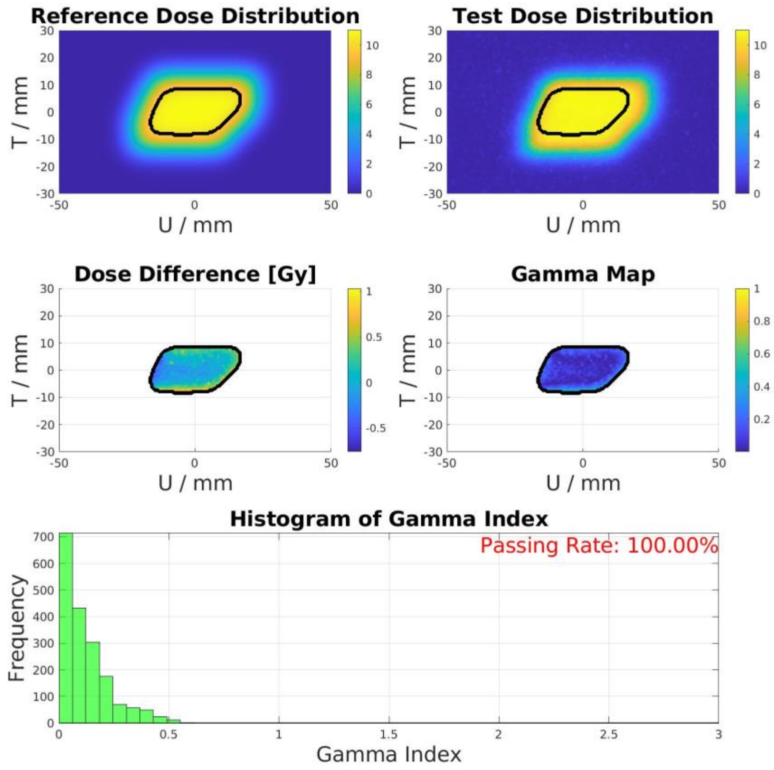
Reference Dose Distribution
Test Dose Distribution
Dose Difference [Gy]
Gamma Map
Histogram of Gamma Index
Passing Rate: 100.00%
T / mm
U / mm
Frequency
Gamma Index

F1

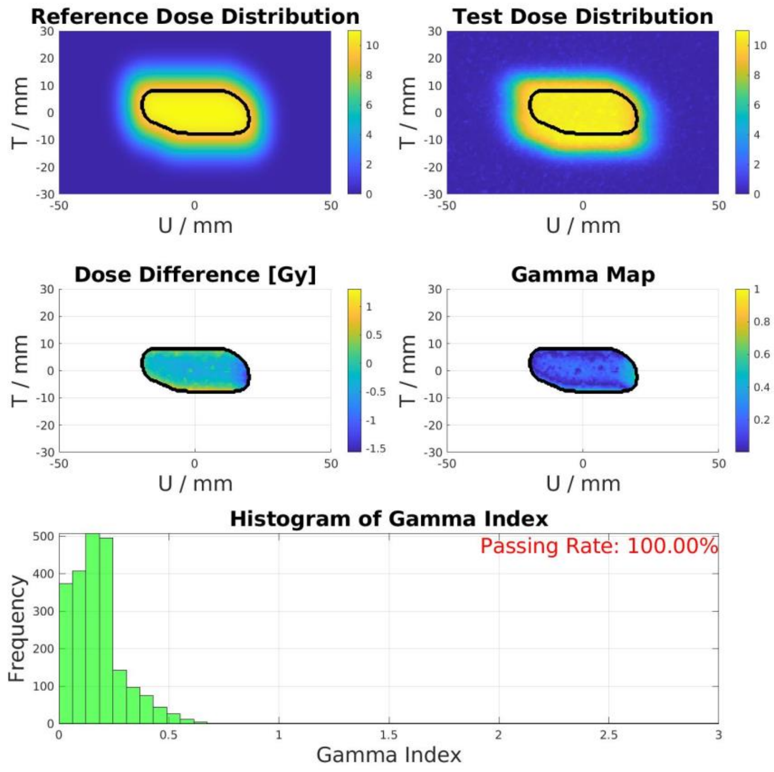
Reference Dose Distribution
Test Dose Distribution
Dose Difference [Gy]
Gamma Map
Histogram of Gamma Index
Passing Rate: 100.00%
T / mm
U / mm
Frequency
Gamma Index

F2

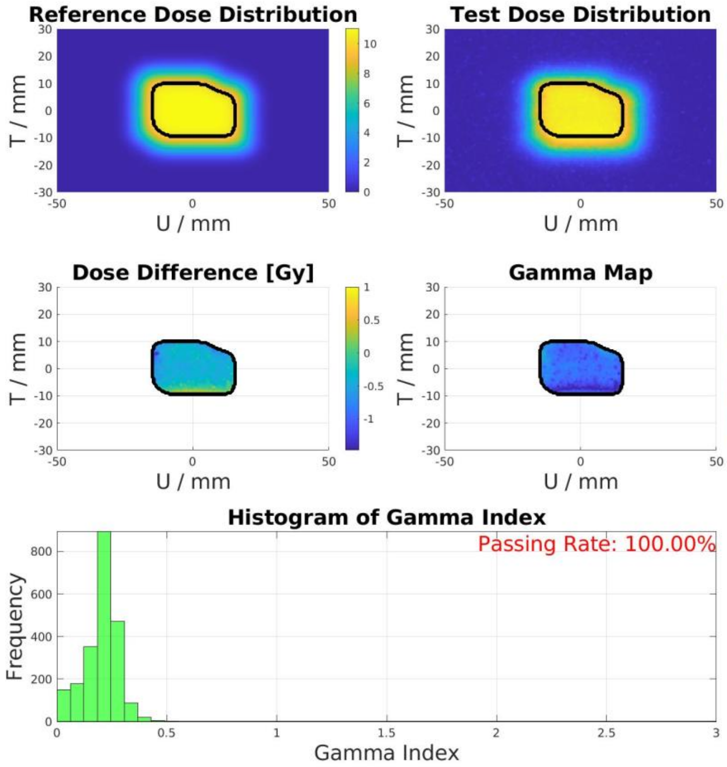
Reference Dose Distribution
Test Dose Distribution
Dose Difference [Gy]
Gamma Map
T / mm
U / mm
Histogram of Gamma Index
Passing Rate: 100.00%
Frequency
Gamma Index

DQA

F0

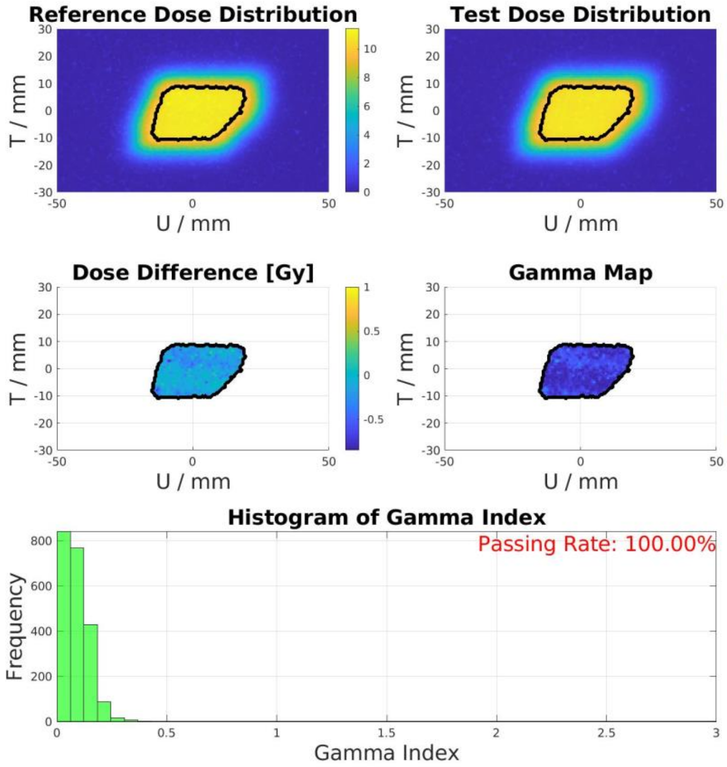
Reference Dose Distribution
Test Dose Distribution
Dose Difference [Gy]
Gamma Map
T / mm
U / mm
Histogram of Gamma Index
Passing Rate: 100.00%
Frequency
Gamma Index

F1

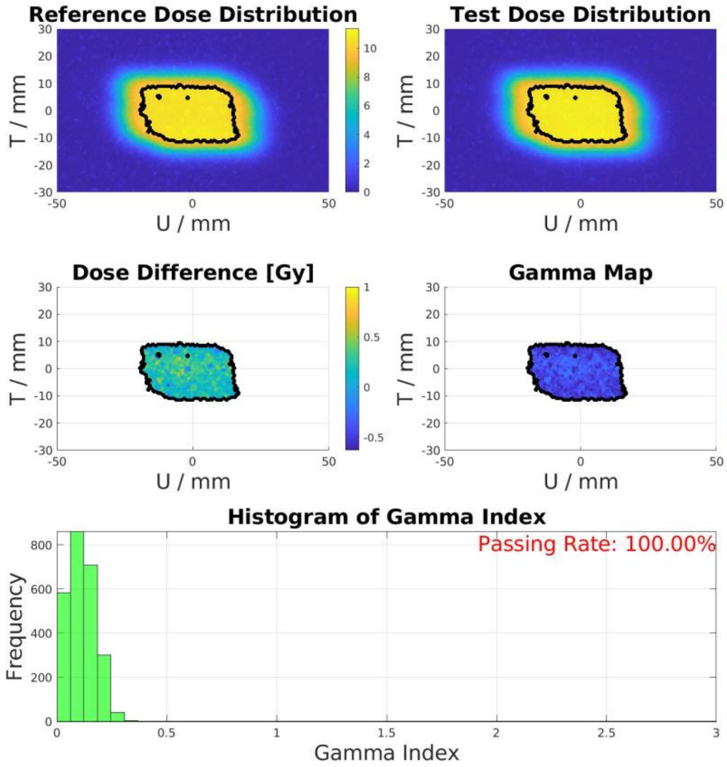
Reference Dose Distribution
Test Dose Distribution
Dose Difference [Gy]
Gamma Map
Histogram of Gamma Index
Passing Rate: 100.00%
T / mm
U / mm
Frequency
Gamma Index

F2

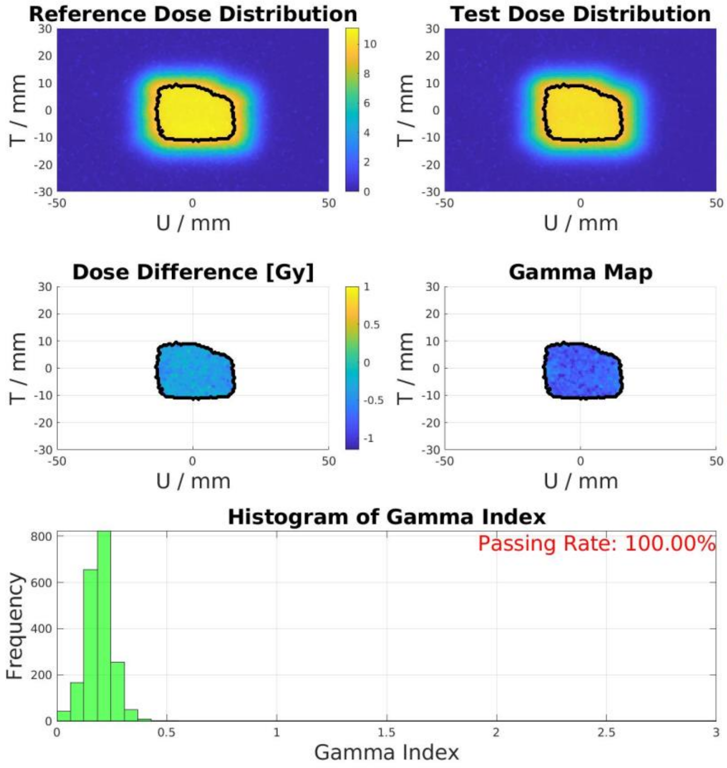
Reference Dose Distribution
Test Dose Distribution
Dose Difference [Gy]
Gamma Map
Histogram of Gamma Index
Passing Rate: 100.00%
T / mm
U / mm
Frequency
Gamma Index

Table A1: Most relevant values logged at 1 kHz sampling rate

Index	Contents	Comments
0	Spot number	–
6	MU per spot	Relevant for dose reconstruction
14	Time	The elapsed time of the beam application
15	External detector counts	Logged data from an external detector wired to our system (typically the micro-diamond detector during QA tests)
16–17	Hall sensor ADC	Converted to magnet current via calibration function (relevant for spot position reconstruction)
18–19	Interlock time/dose limit remaining counts	More on the "debugging"/machine handling side for internal purposes only
